# A DIA‐MS‐based proteomics approach to find potential serum prognostic biomarkers in glioblastoma patients

**DOI:** 10.1002/1878-0261.70068

**Published:** 2025-06-15

**Authors:** Anne Clavreul, François Guillonneau, Odile Blanchet, Hamza Lasla, Audrey Rousseau, Catherine Guette, Alice Boissard, Cécile Henry, Morgan Dhondt, Pascal Jézéquel, Philippe Menei, Jean‐Michel Lemée

**Affiliations:** ^1^ Département de Neurochirurgie CHU d'Angers France; ^2^ Nantes Université, Univ Angers, INSERM, CNRS, CRCI^2^NA France; ^3^ Prot'ICO Proteomics Facility, Institut de Cancérologie de l'Ouest (ICO) Angers France; ^4^ Centre de Ressources Biologiques BB‐0033‐00038, CHU d'Angers France; ^5^ Omics Data Science Unit, Institut de Cancérologie de l'Ouest (ICO) Nantes France; ^6^ SIRIC ILIAD, Institut de Recherche en Santé, Université de Nantes France; ^7^ Département de Pathologie CHU d'Angers France

**Keywords:** blood, glioblastoma, prognosis, proteomics

## Abstract

No blood‐based protein biomarkers are currently available for routine clinical use to determine the prognosis of patients with glioblastoma (GB). We performed data‐independent acquisition mass spectrometry (DIA‐MS)‐based proteomics on 96 presurgical serum samples from patients with GB and 30 serum samples from healthy controls to identify such markers. Among the 622 serum proteins differentially expressed between the GB and control groups, 191 had a |log_2_(fold change)| ≥ 0.58 and an area under the curve ≥ 0.75. An analysis of their prognostic value revealed that high levels of IL1R2 and low levels of CRTAC1 and HRG were associated with poor survival. Multivariate Cox regression analysis identified IL1R2 as an independent prognostic factor for PFS and CRTAC1 as an independent prognostic factor for OS. The concentration of CRTAC1 in serum samples from an independent cohort of short‐ and long‐term survivors of GB (STS and LTS, respectively) by ELISA was shown to be lower in the STS than in the LTS group. CRTAC1, HRG, and IL1R2 could potentially be used to better inform prognosis and predict treatment response in GB patients.

## Graphical abstract

A DIA‐MS‐based proteomics analysis of serum samples from GB patients and healthy controls showed that high levels of IL1R2 and low levels of CRTAC1 and HRG in serum are associated with poor survival outcomes for GB patients. These circulating proteins could serve as biomarkers for the prediction of outcome in patients with GB.

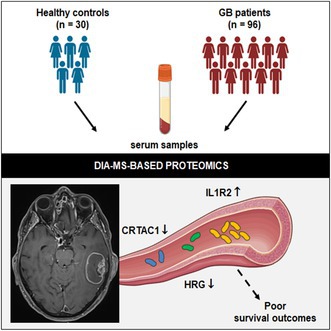

**Abbreviations**
AUC: area under the curve
BBB: blood–brain barrier
BRIF: bioresource research impact factor
DIA‐MS: data‐independent acquisition mass spectrometry
DMNC: density of maximum neighborhood component
EOR: extent of resection
FFPE: formalin‐fixed paraffin‐embedded
GB: glioblastoma
GTR: gross total resection (100%)
IDH: isocitrate dehydrogenase
KPS: Karnofsky performance score
LTS: long‐term survivors
MCC: maximal clique centrality
MGMT: O6‐methylguanine methyltransferase
MIC: minimizing approximated information criteria
OS: overall survival
PCA: principal component analysis
PFS: progression‐free survival
PR: partial resection (< 90%)
ROC: receiver operating characteristic
ROS: reactive oxygen species
SAM: significance analysis of microarray
STR: subtotal resection (≥ 90%)
STS: short‐term survivors
TMZ: temozolomide

## 1 Introduction

Glioblastomas (GB) are the most common and aggressive primary tumors of the central nervous system. The current standard of care for newly diagnosed GB—the Stupp protocol, which includes maximum safe resection followed by radiotherapy plus concomitant and adjuvant temozolomide (TMZ)—can now be combined with tumor treating fields therapy [1, 2]. Nevertheless, patient prognosis remains poor, with a median survival of about 22 months [3].

Significant scientific and technological research is underway to improve GB care [4], including the search for reliable, noninvasive biomarkers for the management of GB patients, which would be useful for assessing prognosis and monitoring response to treatment, detecting true progression at, or even before the first sign of increasing contrast enhancement and facilitating the diagnosis of inoperable tumors. Several genetic and molecular biomarkers in GB tissues have been discovered that facilitate the prediction of prognosis and treatment responses [5]. For example, O6‐methylguanine DNA methyltransferase (*MGMT*) promoter methylation is a potential predictive marker for response to TMZ, and isocitrate dehydrogenase (IDH) mutations have been identified as markers of a favorable prognosis [6, 7]. However, IDH‐mutated GB are now considered to be IDH‐mutated grade 4 astrocytomas, with gene methylation and expression profiles different from those of IDH‐wild‐type GB, and a different prognosis and response to treatment [8, 9].

Blood samples are ideal for assessments of prognosis and the monitoring of treatment response, as they are obtained by less invasive methods than tissue biopsy or cerebrospinal fluid puncture, and repeat samples can be obtained over the course of treatment to evaluate tumor dynamics in real time. We previously showed that preoperative assessments of cellular blood markers, such as neutrophil‐to‐lymphocyte ratio, red blood cell count, and platelet count, can predict the survival outcomes of GB patients treated with the standard therapy [10]. Research efforts are currently focusing on tumor components present in the blood, such as circulating tumor cells, circulating tumor DNA, circulating RNA, extracellular vesicles, and circulating proteins, but to the best of our knowledge, none of these circulating biomarkers has ever been introduced into the routine prognostic monitoring of GB patients [5]. The lack of clinically relevant circulating proteins able to predict survival outcomes and treatment response in GB patients may be explained by the low sensitivity of protein detection by the proteomics technology used in studies and the small size of cohorts of preoperative GB blood samples (< 40) [11, 12, 13, 14].

In this study, we used a data‐independent acquisition mass spectrometry (DIA‐MS)‐based proteomics approach to characterize the serum proteomes of a larger cohort of GB patients (*n* = 96) and healthy controls (*n* = 30). This powerful label‐free technology has been shown to be a suitable next‐generation strategy for high‐throughput quantitative proteomics with a number of potential clinical applications, providing insight into the functional biology of cancers and identifying novel treatment targets and prognostic biomarkers for clinical use [15, 16]. We have already used DIA‐MS to detect differences in protein abundance in tumor and serum samples from short‐ and long‐term survivors of GB (STS and LTS, respectively) [17]. Here, we compared the serum proteomes of healthy controls and patients with GB with available clinical and survival data to identify potential circulating prognostic biomarkers in GB patients. This large set of serum proteomics data associated with clinical data will be of considerable use to the scientific community.

## 2 Methods

### 2.1 Study cohort

This study included 96 patients newly diagnosed with GB between January 2012 and December 2021 at Angers University Hospital. The following inclusion criteria were used: (1) patient aged ≥ 18 years, (2) newly diagnosed unilateral supratentorial GB, (3) GB without immunohistochemical staining for IDH1‐R132H, (4) tumor resected, (5) no intraoperative chemotherapy, and (6) first‐line treatment with complete concomitant chemoradiotherapy according to the Stupp protocol [18]. The number of cycles of subsequent adjuvant chemotherapy with oral TMZ depended on tolerance and radiological response. We also included 30 healthy controls recruited at Angers University Hospital.

### 2.2 Ethics approval and consent to participate

The study was conducted in accordance with the Declaration of Helsinki.

Patients with GB were included in the FGB [19]. The FGB was declared to the French Ministry of Health and Research [declaration number: DC‐2011‐1467, cession authorization number: AC‐2023‐5473, BRIF (bioresource research impact factor) number: BB‐0033‐00093]. The protocols and regulations of the FGB were approved by the CPP OUEST II ethics committee (CB 2012/02, date of approval: 20 December 2011) and the CNIL (“Commission Nationale de l'Informatique et des Libertés,” the French national data protection authority, no. 1476342, date of approval: 10 October 2011). Healthy controls were included in the “CELREMED” biocollection of Angers University Hospital with the approval of the CPP OUEST II ethics committee (CB 2015/02, date of approval: 20 March 2015). The experiments were undertaken with the understanding and written consent of each subject.

### 2.3 Collection of clinical data

Baseline characteristics, such as age, sex, preoperative Karnofsky performance score (KPS), tumor location, blood data before surgery, extent of resection (EOR), *MGMT* methylation status, and Stupp protocol regimen, were collected from medical records. The methylation status of the *MGMT* promoter was assessed by pyrosequencing [20]. Overall survival (OS) was defined as the time from initial surgery to last follow‐up or death. Progression‐free survival (PFS) was measured from initial surgery to the date of first progression.

### 2.4 DIA‐MS and selection of the serum proteins of interest

Blood samples were collected from all 96 GB patients before surgery. Serum aliquots prepared from GB and control blood samples were processed as previously described [17]. Each serum sample (200 ng) was analyzed by LC–MS/MS with a nanoElute UHPLC system (Bruker Daltonik GmbH, Bremen, Germany) based on an Aurora series reverse‐phase C_18_ column (25 cm × 75 μm internal diameter, 1.6 μm C_18_, IonOpticks, Fitzroy, Australia) heated to 50°C and coupled to a TimsTOF Pro2 (Bruker Daltonik GmbH). A linear gradient of 2–35% mobile phase B (0.1% formic acid in acetonitrile) over 60 min was used with 0.1% formic acid in milliQ‐grade H_2_O as mobile phase A. The total run time, including a ramping up of phase B from 35 to 95% to clean the column and prepare for the next sample, was 90 min. Measurements were acquired in DIA‐PASEF mode (data‐independent acquisition parallel accumulation serial fragmentation). We analyzed 200 ng of peptides from each sample over a 60‐min gradient. The default *m/z* range was 400–1201, with an IM range of 0.6–1.43 1/K0 [V s·cm^−2^], corresponding to an estimated cycle time of 1.80 s. Default settings were also used for the DIA‐PASEF windows and collision energy, with a base of 0.85 1/K0 [V s·cm^−2^] set at 20 eV and 1.30 1/K0 [V s·cm^−2^] set at 59 eV. TIMS and mass calibration were performed linearly with three calibrating ions at 622, 922, and 1222 *m/z* (Agilent Technologies, Les Ulis, France) at different ranges to match the Δ1/K0 of the corresponding set of ranges. Mass spectrometry data were analyzed with DIA‐NN v.1.8.1, by searches against the reviewed Human Uniprot database (retrieved 4/21), with the software in library‐free mode. The match‐between‐runs feature and intersample normalization were used for all analyses, and the output (precursors) was filtered to keep the false discovery rate below 1%. Retention time alignment and correction for mass accuracy were performed automatically. The data were further log_2_‐transformed. For each protein with less than 30% missing values overall across samples, data imputation was performed with one‐fifth the lowest recorded value for expression used to replace missing values so that statistical tests could be performed. Proteins with more than 30% missing values across samples were excluded from the analysis. Principal component analysis (PCA) was used to identify outliers. The significance analysis of microarray (SAM) method was used with the “samr” R package to identify proteins displaying significant differential expression between the GB and control groups. Proteins with a SAM *Q*‐value < 0.05 were considered to display significant differential expression. The diagnostic performance of the serum proteins was assessed by plotting the receiver operating characteristic (ROC) curve and calculating the area under the curve (AUC) with the “survivalROC” R package. An AUC value ≥ 0.75 was considered significant. Proteins displaying significant differential expression with a |log_2_(FC)| ≥ 0.58 and an AUC ≥ 0.75 were considered to be proteins of interest.

### 2.5 Functional enrichment, module and hub protein analysis for the serum proteins of interest

The biological functions and potential signaling pathways of the serum proteins of interest were analyzed with Metascape (https://metascape.org) [21]. Protein–protein interaction (PPI) network analysis was performed with the STRING database version 12.0 [22]. A confidence score of ≥ 0.40 was selected for the construction of the PPI network in Cytoscape version 3.9.0. We used Molecular Complex Detection (MCODE; version 1.5.1) [23] to identify the most significant module in the PPI network. MCODE scores > 3 and a number of nodes ≥ 5 were set as the cutoff criteria with the default parameters (degree cutoff ≥ 2, node score cutoff ≥ 2, k‐core ≥ 2 and max depth = 100).

### 2.6 Analysis of the prognostic value of the serum proteins of interest

Univariate Cox regression analyses were performed to assess the prognostic value of the serum proteins of interest based on the survival data of the 96 GB patients. Proteins were analyzed as both continuous and dichotomous variables. Dichotomization was achieved by a median split or use of an optimal cutoff set according to the maximally selected rank statistics from the “maxstat” R package. The minimizing approximated information criteria (MIC) method was implemented in the “coxphMIC” R package [24] to select serum proteins of potential prognostic value. These potential prognostic markers were then evaluated in a multivariate Cox regression analysis including relevant clinical information, such as age, sex, KPS, and TMZ consolidation treatment, unless these variables were correlated. Survival curves were plotted according to the Kaplan–Meier method and were compared by the log‐rank test.

### 2.7 Immunohistochemistry (IHC) for CRTAC1

IHC was performed for CRTAC1 (cartilage acidic protein 1) on 4‐μm sections of formalin‐fixed paraffin‐embedded (FFPE) tissue blocks with an automated Leica BOND III (Leica Biosystems, Nanterre, France) in accordance with the manufacturer's instructions. Fourteen FFPE tissue blocks from STS (*n* = 7, OS ≤ 12 months) and LTS with GB (*n* = 7, OS ≥ 30 months) whose paired serum samples were analyzed by DIA‐MS were selected for IHC. The anti‐CRTAC1 antibody (Proteintech, Planegg‐Martinsried, Germany) was used at a dilution of 1/50 in BOND Primary Antibody Diluent (Leica) for EDTA antigen retrieval and was visualized with BOND Polymer Refine Detection (Leica). Digital images were captured with an Aperio CS2 scanner (Leica) fitted with a 20x objective and analyzed with the Aperio ImageScope software v12.3.2.8009 (Leica). A semiquantitative analysis of IHC images was performed with the Fiji software. The percent positivity was calculated as the number of positive pixels divided by the total number of pixels in the area analyzed, multiplied by 100. Six tumor areas of identical size were analyzed in each section, and the mean percent positivity was calculated.

### 2.8 Enzyme‐linked immunosorbent assay (ELISA) for CRTAC1

The concentration of CRTAC1 was also determined in serum samples from an independent cohort of STS (*n* = 12) and LTS with GB (*n* = 13), with a commercial ELISA kit for CRTAC1 (EH133RB; Invitrogen, Fisher Scientific, Illkirch, France) used according to the manufacturer's instructions. Serum samples from 12 healthy controls used in the DIA‐MS analysis were also included. CRTAC1 concentration was calculated by comparison with a standard curve. The samples were evaluated in duplicate.

### 2.9 Statistical analyses

Statistical analyses were performed with the r software (version 4.1.0; https://www.r‐project.org). The *P*‐values were corrected for multiple comparisons (generating *Q*‐values). Values of *P* < 0.05 or *Q* < 0.05 were considered statistically significant. In addition to the tests described above, differences between groups were assessed with the Mann–Whitney *U* test or the Kruskal–Wallis test for quantitative variables. The correlation of serum levels of potential prognostic proteins with hematological parameters was analyzed by calculating Pearson's correlation coefficient.

## 3 Results

### 3.1 Identification of serum proteins of interest

We analyzed 126 serum samples (GB = 96, Control = 30) by DIA‐MS. The baseline characteristics of the 96 selected GB patients and healthy controls are shown in Table 1. In total, 1305 proteins (963 with < 30% missing values) were identified in serum samples (Table S1). PCA identified no outliers among the serum samples. We identified 622 serum proteins as displaying significant differential expression between the GB and control groups (Table S1). These proteins included 191 with a |log_2_(FC)| ≥ 0.58 and an AUC ≥ 0.75 (Table S2), which we considered to be proteins of interest. We found that 89 serum proteins of interest were upregulated in the GB group and 102 were upregulated in the control group (Table S2).

**Table 1:** Demographic and clinical characteristics of healthy controls and patients with GB treated with a first‐line Stupp's regimen.

Healthy controls	
Number	30 (100%)
Median (range)	70 (42–80)
Sex	
Male	15 (50%)
Female	15 (50%)
Patients with GB	
Number	96 (100%)
Age (years)
Median (range)	63 (36–81)
Sex	
Male	66 (69%)
Female	30 (31%)
Preoperative KPS (%)	
≤ 80	21 (22%)
> 80	75 (78%)
Tumor laterality	
Right	51 (53%)
Left	45 (47%)
Extent of tumor	
Unilobar	51 (53%)
Multilobar	45 (47%)
EOR	
PR	11 (11%)
STR	27 (28%)
GTR	58 (60%)
*MGMT* methylation status	
Without methylation	12 (13%)
With methylation	12 (13%)
Unknown	72 (75%)
TMZ consolidation	
< 6 cycles	60 (63%)
≥ 6 cycles	35 (36%)
Unknown	1 (1%)
Survival outcome	
PFS	
Median (months) [95% CI]	7.5 [6.5–8.8]
OS	
Median (months) [95% CI]	17.1 [14.4–19.1]

EOR, extent of resection; GTR, gross total resection (100%); KPS, Karnofsky performance score; *MGMT*, O6‐methylguanine methyltransferase; OS, overall survival; PFS, progression‐free survival; PR, partial resection (< 90%); STR, subtotal resection (≥ 90%); TMZ, temozolomide.

### 3.2 Functional enrichment analysis on the serum proteins of interest and identification of key modules

**Fig. 1:**
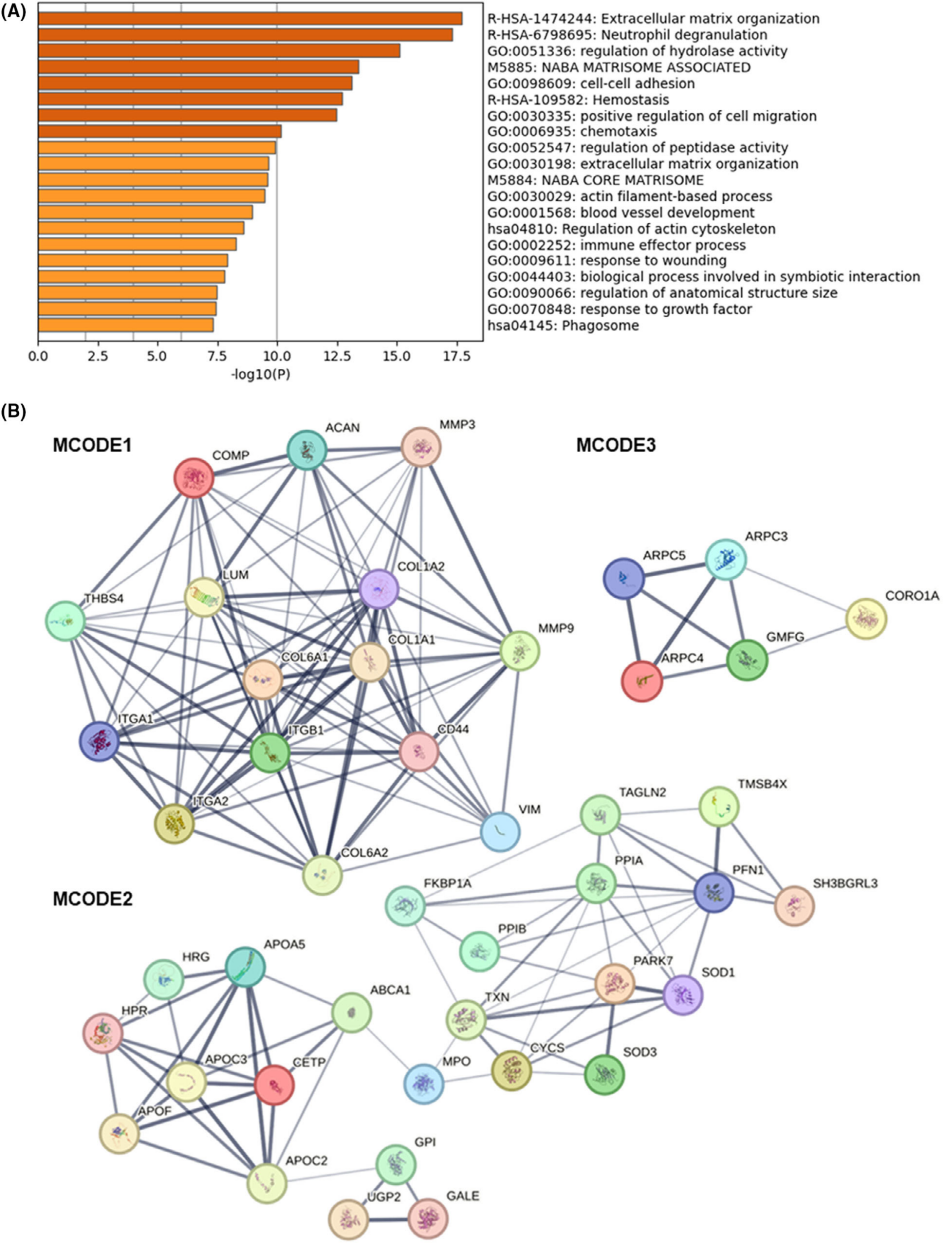
Functional enrichment analysis and identification of key modules. (A) Metascape bar graph of functional term enrichment for the 191 serum proteins of interest (color‐coded according to *P*‐values). (B) Identification of the three most significant modules in the PPI network for the 191 serum proteins of interest with MCODE in Cytoscape (MCODE1: score = 12.3, nodes = 15; MCODE2: score = 5.4, nodes = 24; MCODE3: score = 4, nodes = 5). GB, glioblastoma; PPI, protein–protein interaction.

Metascape analysis of the 191 serum proteins of interest indicated that they were associated with 13 gene ontology (GO) biological processes, three reactome gene sets, two canonical pathways, and two KEGG pathways (Fig. 1A). The terms with the top three best *P*‐values were as follows: extracellular matrix organization (R‐HSA‐1474244), neutrophil degranulation (R‐HSA‐6798695), and regulation of hydrolase activity (GO:0051336) (Fig. 1A). PPI network analysis on the 191 serum proteins of interest identified 178 nodes and 583 edges. Three significant modules were extracted with the MCODE plugin tools of Cytoscape applied to the PPI network, identifying 44 hub proteins (Fig. 1B and Table S3). The terms with the best *P*‐value for each MCODE indicated by the Metascape analysis were: extracellular matrix organization (R‐HSA‐1474244), plasma lipoprotein assembly, remodeling, and clearance (R‐HSA‐174824) and Arp2/3 protein complex (CORUM:27) for MCODE1, MCODE2, and MCODE3, respectively.

### 3.3 Prognostic value of the serum proteins of interest and selection of potential prognostic proteins

**Fig. 2:**
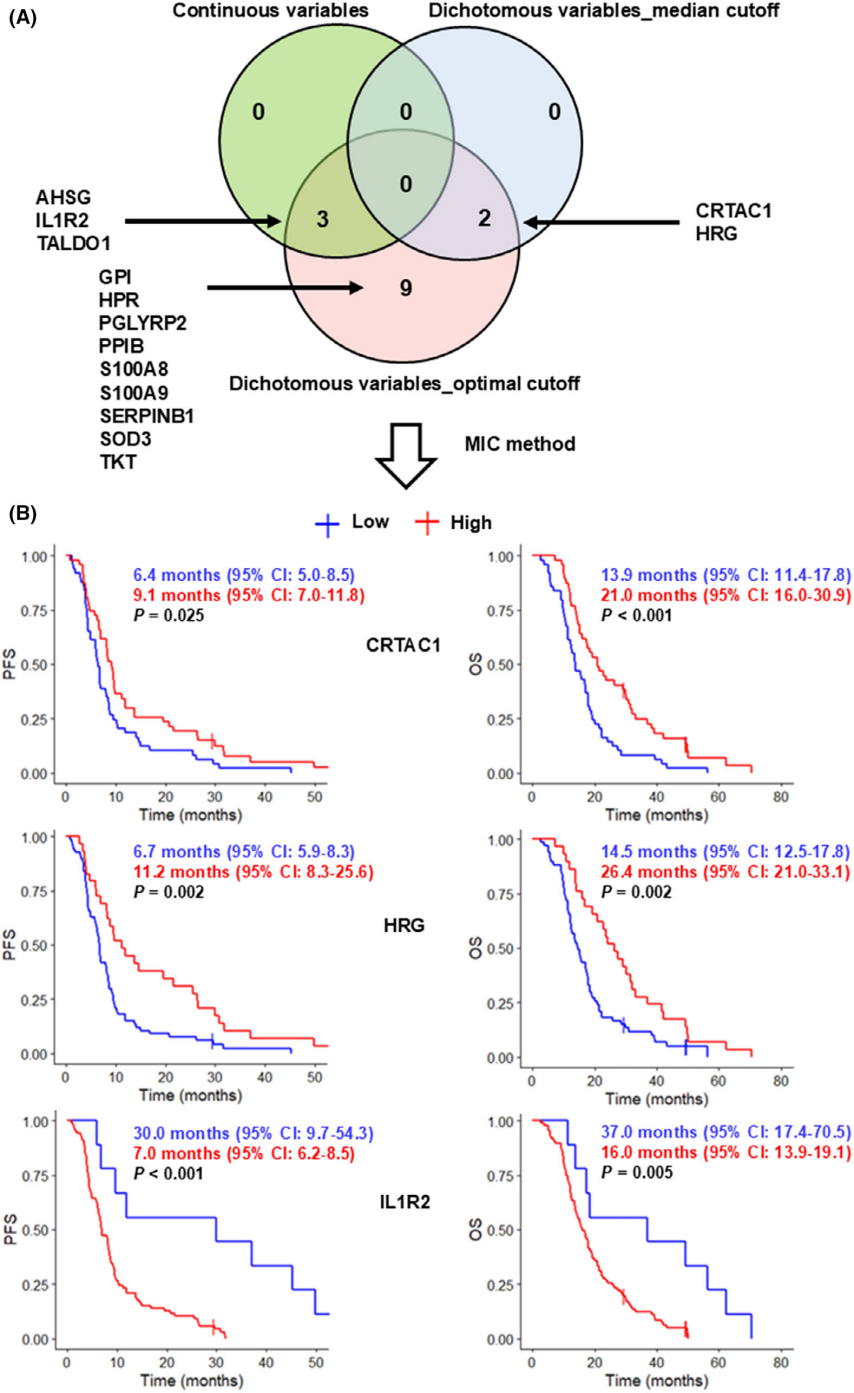
Identification of potentially prognostic proteins in the serum of GB patients (*n* = 96). (A) Venn diagram and MIC method identifying the three potentially prognostic serum proteins. The following criteria were used to select prognostic serum proteins: (1) proteins differentially expressed between the GB and control groups with a SAM *Q*‐value < 0.05, |log_2_(FC)| ≥ 0.58 and AUC ≥ 0.75, (2) proteins consistently associated with PFS and OS in univariate Cox regression analysis performed by at least two methods (continuous variables or dichotomous variables defined according to the median or the optimal cutoff), (3) proteins quantified in all GB samples, (4) proteins showing a concordant pattern (i.e., direction of regulation) between differential analysis and prognostic analysis. Five proteins met these criteria: AHSG, CRTAC1, HRG, IL1R2, and TALDO1; the MIC method selected CRTAC1, HRG, and IL1R2 as potential prognostic biomarkers. (B) Kaplan–Meier curves for CRTAC1, HRG, and IL1R2. The log‐rank test was performed to determine the statistical significance of survival differences (*P* < 0.05). GB, glioblastoma; OS, overall survival; PFS, progression‐free survival; MIC, minimizing approximated information criterion.

The results of univariate Cox regression analysis on the 191 serum proteins of interest differed according to the treatment of these proteins as continuous variables or as dichotomous variables defined according to the median or the optimal cutoff (Tables S4 and S5). We decided to select candidate prognostic biomarkers according to the following criteria: prognostic value for PFS and OS in at least two methods of univariate Cox regression analysis, quantification of expression in all GB samples, and concordant pattern (i.e., direction of regulation) between differential analysis and prognostic analysis. Five proteins met these criteria: AHSG (A2–Heremans–Schmid glycoprotein), CRTAC1, HRG (histidine‐rich glycoprotein), IL1R2 (interleukin 1 receptor type 2), and TALDO1 (transaldolase 1) (Fig. 2A). Nine other serum prognostic biomarkers were excluded by this method because they were associated with PFS and OS only when considered as dichotomous variables with the optimal cutoff: GPI (glucose‐6‐phosphate isomerase), HPR (haptoglobin‐related protein), PGLYRP2 (peptidoglycan recognition protein 2), PPIB (peptidylprolyl isomerase B), S100A8 (S100 calcium‐binding protein A8), S100A9, SERPINB1 (serpin family B member 1), SOD3 (superoxide dismutase 3), and TKT (transketolase) (Fig. 2A). The MIC method applied to the five selected proteins identified CRTAC1, HRG, and IL1R2 as potential prognostic biomarkers (Fig. 2A).

Patients with low levels of CRTAC1, low levels of HRG, or high levels of IL1R2 in the serum had a significantly poorer PFS and OS (Fig. 2B). Multivariate Cox regression analysis was used to assess the independent predictive value of serum levels of CRTAC1, HRG, and IL1R2 for PFS and OS. Serum IL1R2 level remained an independent prognostic factor for PFS, and serum CRTAC1 level remained an independent prognostic factor for OS after adjustment for other variables, including age, sex, KPS, and TMZ (Table S6).

### 3.4 Verification of the prognostic value of CRTAC1

**Fig. 3:**
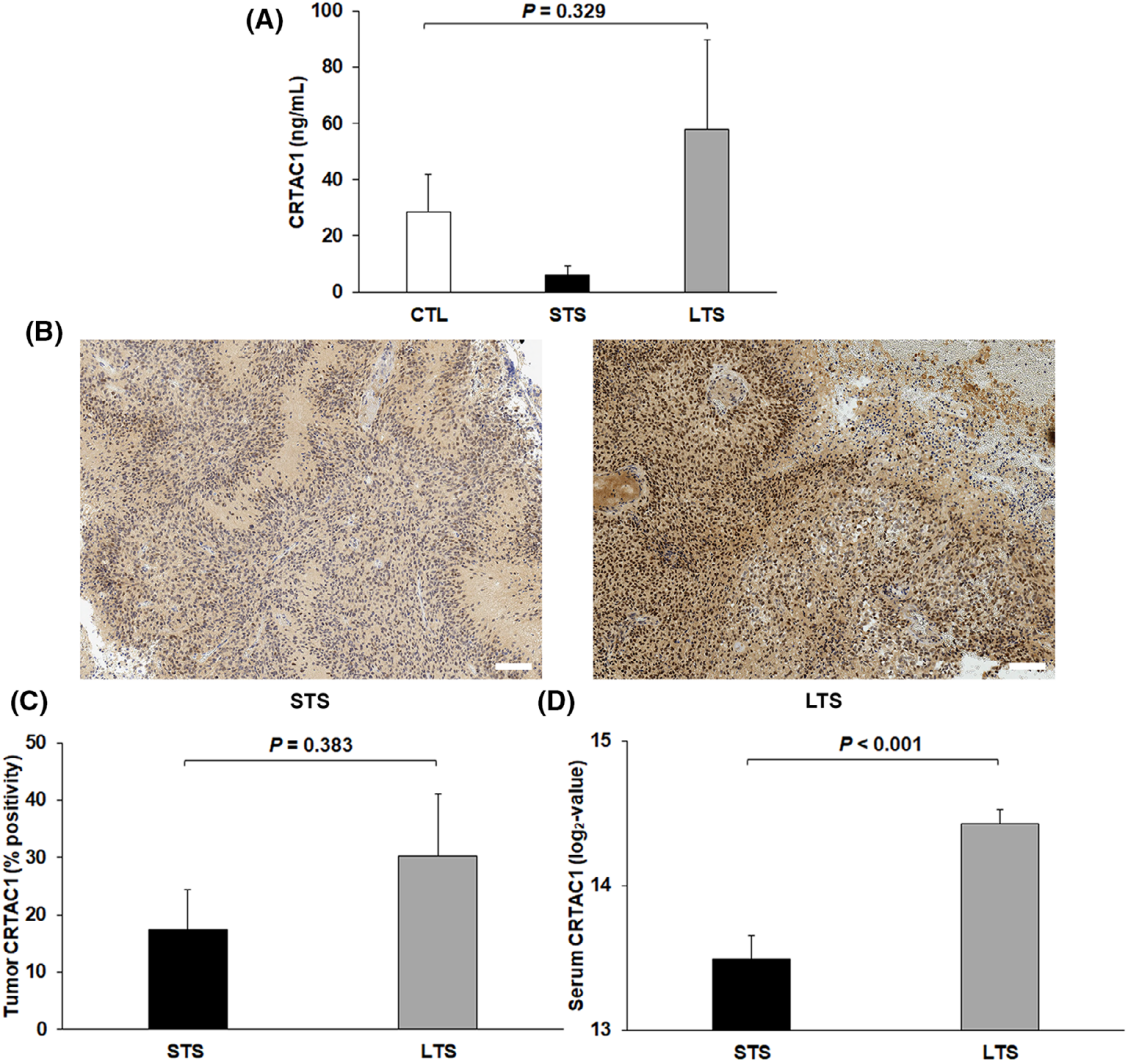
Verification of the prognostic value of CRTAC1. (A) CRTAC1 levels, as assessed by ELISA, in serum samples from healthy controls (*n* = 12) and an independent cohort of STS (*n* = 12) and LTS with GB (*n* = 13). There was no significant difference between the groups in a Kruskal–Wallis test (mean ± SEM, *P* = 0.329). (B) Example of IHC images of CRTAC1 expression in tumor samples from STS (*n* = 7) and LTS (*n* = 7) with GB. The scale bar corresponds to 100 μm. (C) and (D) CRTAC1 levels in paired serum and tumor samples from STS (*n* = 7) and LTS with GB (*n* = 7). Serum CRTAC1 levels, as determined by DIA‐MS, were significantly lower in the STS group than in the LTS group (mean ± SEM, *P* < 0.001, Mann–Whitney *U* test) (D). Tumor CRTAC1 levels, as determined by IHC, were also lower in the STS group than in the LTS group, but this difference was not significant (mean ± SEM, *P* = 0.383, Mann–Whitney *U* test) (C). CTL, healthy controls; GB, glioblastoma; IHC, immunohistochemistry; LTS, long‐term survivors; SEM, standard error of the mean; STS, short‐term survivors.

Serum CRTAC1 level was identified as an independent prognostic factor for OS. We therefore checked, by ELISA, the concentrations of this protein in serum samples from healthy controls and an independent cohort of STS and LTS with GB. CRTAC1 concentrations were higher in the LTS and healthy control groups than in the STS group, but this difference was not significant in a Kruskal–Wallis test (*P* = 0.329) (Fig. 3A). The commercial ELISA kit used may not be suitable for serum CRTAC1 quantification, as many samples had CRTAC1 levels below the minimum detectable concentration (1.23 ng·mL^−1^): control group = 41.7%, STS group = 66.7%, and LTS group = 61.5%. We also investigated the expression of CRTAC1 in tissues by IHC staining on tumor specimens from STS and LTS whose paired serum samples were analyzed by DIA‐MS. CRTAC1 is expressed in tumor cells and in the extracellular matrix (Fig. 3B). A semiquantitative analysis of IHC images indicated that the percent positivity for CRTAC1 was lower in STS tissues (17.4 ± 6.9%) than in LTS tissues (30.2 ± 10.9%), but this difference was not significant in a Mann–Whitney *U* test (*P* = 0.383) (Fig. 3C). CRTAC1 levels in paired serum samples, as determined by DIA‐MS, were also lower in the STS group than in the LTS group (*P* < 0.001, Mann–Whitney *U* test) (Fig. 3D).

### 3.5 Analysis of the association between CRTAC1, HRG, and IL1R2 levels and hematological parameters

We assessed the association of CRTAC1, HRG, and IL1R2 with hematological parameters (Table S7). Total white blood cell and neutrophil counts were significantly positively correlated with IL1R2 levels (*R* = 0.37; *Q* = 0.005, *R* = 0.38; *Q* = 0.003, respectively) and negatively correlated with CRTAC1 (*R* = −0.57; *Q* < 0.001, *R* = −0.52; *Q* < 0.001, respectively) and HRG (*R* = −0.39; *Q* = 0.002, *R* = −0.38; *Q* = 0.003, respectively) levels.

## 4 Discussion

DIA‐MS analysis identified 622 serum proteins displaying significant differential expression between the GB and control groups, including 191 proteins of interest with a |log_2_(FC)| ≥ 0.58 and an AUC ≥ 0.75. Functional analysis of the 191 proteins of interest indicated that they were associated with several enriched terms, the most significant of which were extracellular matrix organization, neutrophil degranulation, and regulation of hydrolase activity. Arora et al. identified 40 proteins with a differential abundance in serum samples from GB patients with the 4‐plex iTRAQ methodology [13]. In our study, 20 of these proteins displayed concordant dysregulation between GB and control sera, and seven were among the 191 proteins of interest: AHSG, C1QA (complement C1q A chain), HRG, LUM (lumican), PGLYRP2, S100A8, and S100A9.

An analysis of the prognostic value of the 191 serum proteins of interest identified three potential circulating biomarkers: CRTAC1, HRG, and IL1R2. Serum levels of CRTAC1 and HRG were lower in GB patients than in healthy controls, and low levels of these proteins were associated with a poor PFS and OS. By contrast, serum levels of IL1R2 were higher in GB patients than in healthy controls, and high levels of this protein were associated with a poor PFS and OS. IL1R2 was found to be an independent prognostic factor for PFS, and CRTAC1 was identified as an independent prognostic factor for OS. Assessing potential correlations between the levels of these serum prognostic biomarkers, chromosomal or genetic alterations in GB tissues, and the MGMT methylation status was not feasible due to significant missing data in the cohort. At our center, these analyses are not mandatory in pathology reports, as they have limited relevance to first‐line clinical decision‐making. Moreover, the lack of large‐scale serum proteomics data for GB associated with clinical data also prevented the use of such datasets to support the prognostic potential of CRTAC1, HRG, and IL1R2. To overcome this, we analyzed the concentration of CRTAC1 in serum samples from an independent cohort of STS and LTS with GB using ELISA, as this protein has been identified as an independent prognostic factor for OS. We observed that the levels of this protein were lower in the STS group than in the LTS group. This is consistent with our previous DIA‐MS‐based proteomics analysis comparing serum samples from STS and LTS with GB [17] in which serum levels of CRTAC1 and HRG were lower in the STS group than in the LTS group, whereas serum levels of IL1R2 were higher in the STS group. However, the sample sizes were too small for ELISA and DIA‐MS analyses to have sufficient power to demonstrate a significant difference. The low proportion of LTS with GB makes it difficult to obtain a large cohort of biological samples. Eleven other proteins—AHSG, GPI, HPR, PGLYRP2, PPIB, S100A8, S100A9, SERPINB1, SOD3, TALDO1, and TKT—were also identified as interesting serum prognostic biomarkers but were not selected with the filtering criteria used. Two of these proteins, S100A8 and AHSG, have already been reported to be of prognostic value in GB patients [13, 25]. The levels of malate dehydrogenase 1 (MDH1) and ribonuclease inhibitor 1 (RNH1)—two serum proteins associated with reactive oxygen species (ROS) detoxification that we previously found significantly upregulated in the STS group relative to the LTS group [17]—did not differ significantly between the GB and control groups in this study, highlighting an obstacle to the use of these protein biomarkers for prognostic monitoring due to the difficulty of defining relevant expression thresholds [17]. GPI, TALDO1, and TKT, which are associated with ROS detoxification, are possible alternatives to MDH1 and RNH1.

Our results are consistent with published data on the involvement of CRTAC1, HRG, and IL1R2 in oncogenesis. IL‐1R2, an IL‐1 decoy receptor, is overexpressed in various cancers (tissues and/or serum), including breast, gastric, and pancreatic cancers, and is linked to a poor prognosis [26, 27, 28]. HRG, a protein mainly produced in the liver but can also be synthesized by monocytes and macrophages, has been shown to slow tumor growth in mouse models of various cancers, including GB [29, 30]. Lower levels of HRG in the blood were associated with a poorer prognosis in ovarian and pancreatic cancers [31]. Serum HRG level was strongly correlated with the serum level of AHSG, another protein produced in the liver for which low levels are known to be associated with poor OS in GB patients [25]. CRTAC1, an extracellular matrix protein initially isolated from human chondrogenic tissue [32], was found to be present at lower levels in the plasma of glioma patients compared with nonglioma patients, and in glioma tissue compared with tumor‐free brain tissue [33]. Low tumor levels of CRTAC1 are predictive of a poor prognosis in various cancers, including bladder cancer, gastric cancer, lung cancer, and low‐grade glioma [34, 35, 36, 37]. Consistently, we observed that CRTAC1 levels tended to be lower in paired tumor and serum samples from STS than in those from LTS.

CRTAC1, HRG, and IL1R2 are not cancer‐specific biomarkers, but they are broadly associated with various diseases. For example, IL1R2 is a blood biomarker of several inflammatory diseases such as Dengue, sepsis, and rheumatoid arthritis, but the relationship between circulating levels and disease prognosis or severity is variable [26]. HRG is a blood prognostic biomarker in patients with sepsis and community‐acquired pneumonia, with low levels of HRG being associated with greater disease severity [38, 39]. Elevated blood CRTAC1 levels are associated with poor outcomes in patients with acute ischemic stroke and osteoarthritis [40, 41].

The detailed mechanisms of action of CRTAC1, HRG, and IL1R2 in cancer progression are not yet fully established. The upregulation of IL1R2 has been shown to orchestrate an immunosuppressive tumor microenvironment, promote tumor proliferation and invasion, and activate the expression of angiogenic factors [26, 28, 42]. Several studies have reported that CRTAC1 upregulation inhibits tumor cell proliferation, invasion, and migration, as well as glycolysis and angiogenesis, and increases chemosensitivity [37, 43, 44, 45]. HRG has been described to inhibit tumor growth and metastasis by promoting M1 polarization of tumor‐associated macrophages and tumor vessel normalization [30, 46]. We observed that serum CRTAC1 and HRG levels were inversely correlated with circulating neutrophil counts. We and others have previously shown that circulating neutrophil levels are major determinants of immunosuppression, progression, and treatment resistance in GB [10, 47, 48]. This highlights that CRTAC1 and HRG could also mediate their antitumor effects by decreasing the recruitment of neutrophils. Consistently, Yin et al. showed that HRG inhibited liver cancer metastasis to the lung by reducing the recruitment of neutrophils and the production of neutrophil extracellular traps [49]. Similarly, You et al. found an inverse correlation between CRTAC1 expression and neutrophil infiltration in patients with bladder cancer [50].

The underlying mechanisms which control the release of CRTAC1, HRG, and IL1R2 into the bloodstream of GB patients require further investigation. The correlation of CRTAC1 levels between paired tumor and serum samples indicates that the low levels of circulating CRTAC1 may be due to reduced production of this protein by GB cells. The factors contributing to CRTAC1 downregulation in GB tissues remain unclear. Mutations in the promoter region of *CRTAC1* have been suggested [33]. Hypermethylation of the *CRTAC1* promoter could also be responsible for its reduced expression (UALCAN portal, http://ualcan.path.uab.edu) [51, 52]. Consistent with this, *CRTAC1* promoter hypermethylation has been observed in bladder cancer and lung adenocarcinoma [37, 44]. Regarding HRG, its low blood levels in GB patients may result from overall liver function deterioration or increased HRG degradation by tumor cells. In accordance with this, IHC analysis of GB tissues rarely detected HRG in tumor cells (Human Protein Atlas, https://www.proteinatlas.org) [53]. The high blood levels of IL1R2 in GB patients may result from increased secretion by GB cells, which are able to express this protein (Human Protein Atlas). This may also result from increased secretion by neutrophils, which are reported to highly express IL1R2. Our findings, which show that high levels of IL1R2 in preoperative serum samples from GB patients are associated with higher counts of circulating neutrophils, support this hypothesis.

## 5 Conclusion

DIA‐MS‐based proteomics identified three serum proteins—CRTAC1, HRG, and IL1R2—that are differentially expressed between the GB and control groups and may have potential prognostic value. Further studies are now needed to understand the mechanisms regulating the levels of these proteins in the bloodstream. As these serum proteins are not specific to GB, a machine‐learning model that integrates their serum levels with clinical, genomic, and imaging data will be essential to utilize them as reliable prognostic biomarkers for GB patients.

## Conflict of interest

The authors declare no conflict of interest.

## Author contributions

AC conceptualized the study, performed the data analysis, statistically analyzed the data, wrote the original draft of the manuscript, and reviewed and edited the manuscript; FG conceptualized the study, processed the quantitative DIA‐MS data, and reviewed and edited the manuscript; OB contributed to storage and provision of tumor and serum samples; HL performed the data analysis, statistically analyzed the data, and reviewed and edited the manuscript; AR performed the diagnostic analysis, and reviewed and edited the manuscript; CG processed the quantitative DIA‐MS data, and reviewed and edited the manuscript; AB and CH contributed to sample preparation and DIA‐MS analysis; MD contributed to sample preparation and IHC analysis; PJ statistically analyzed the data, and reviewed and edited the manuscript; PM was the coordinator of the FGB; J‐ML conceptualized the study, statistically analyzed the data, and reviewed and edited the manuscript. All the authors have read and agreed to the submission of the published version of the manuscript.

## Peer review

The peer review history for this article is available at https://www.webofscience.com/api/gateway/wos/peer‐review/10.1002/1878‐0261.70068.

## Supporting information


**Table S1.** SAM results for the identification of serum proteins displaying significant differential expression between the GB and control groups.
**Table S2.** Serum proteins of interest with a |log_2_(FC)| ≥ 0.58 and an AUC ≥ 0.75. Sens: GB vs. controls.
**Table S3.** List of the 44 hub proteins.
**Table S4.** Univariate Cox regression analysis of PFS for the 191 serum proteins of interest based on a serum proteome dataset for 96 GB patients treated with a first‐line Stupp's regimen.
**Table S5.** Univariate Cox regression analysis of OS for the 191 serum proteins of interest based on a serum proteome dataset for 96 GB patients treated with a first‐line Stupp's regimen.

**Table S6.** Multivariate Cox regression analysis of factors associated with PFS and OS in 96 GB patients treated with a first‐line Stupp's regimen.
**Table S7.** Relationship between serum prognostic protein levels and hematological parameters, assessed with Pearson's chi‐squared test.

## Data Availability

The mass spectrometry proteomics data have been deposited in the ProteomeXchange Consortium database via the PRIDE [54] partner repository with the dataset identifier PXD045467. The clinical datasets are available from the corresponding author under the authorization of the delegation for clinical research and innovation (DRCI, CHU, Angers) and the ICO (Angers).
