# Supplementary material for: A DIA‐MS‐based proteomics approach to find potential serum prognostic biomarkers in glioblastoma patients
Source: Mol Oncol. 2025 Jun 15;19(9):2686–99. doi: 10.1002/1878-0261.70068 (PMC12420366; doi:10.1002/1878-0261.70068)
Supplement: Supplementary file 2 — Table S6. Multivariate Cox regression analysis of factors associated with PFS and OS in 96 GB patients treated with a first‐line Stupp's regimen. Table S7. Relationship between serum prognostic protein levels and hematological parameters, assessed with Pearson's chi‐squared test. [file MOL2-19-2686-s001.docx]

**A DIA-MS-based proteomics approach to find potential serum prognostic biomarkers in glioblastoma patients**

Anne Clavreul, François Guillonneau, Odile Blanchet, Hamza Lasla, Audrey Rousseau, Catherine Guette, Alice Boissard, Cécile Henry, Morgan Dhondt, Pascal Jézéquel, Philippe Menei, Jean-Michel Lemée

**Supplementary Tables:**

**Table S1.** SAM results for the identification of serum proteins displaying significant differential expression between the GB and control groups, uploaded as separate excel file.

**Table S2.** Serum proteins of interest with a |log_2_(FC)| ≥ 0.58 and an AUC ≥ 0.75. Sens: GB vs. controls, uploaded as separate excel file.

**Table S3.** List of the 44 hub proteins, uploaded as separate excel file.

**Table S4.** Univariate Cox regression analysis of PFS for the 191 serum proteins of interest based on a serum proteome dataset for 96 GB patients treated with a first-line Stupp's regimen, uploaded as separate excel file.

**Table S5.** Univariate Cox regression analysis of OS for the 191 serum proteins of interest based on a serum proteome dataset for 96 GB patients treated with a first-line Stupp's regimen, uploaded as separate excel file.

**Table S6.** Multivariate Cox regression analysis of factors associated with PFS and OS in 96 GB patients treated with a first-line Stupp's regimen.

**Table S7.** Relationship between serum prognostic protein levels and hematological parameters, assessed with Pearson's chi-squared test.

**Table S6.** Multivariate Cox regression analysis of factors associated with PFS and OS in 96 GB patients treated with a first-line Stupp's regimen. CI, confidence interval; HR, hazard ratio, KPS, Karnofsky performance score; OS, overall survival; PFS, progression-free survival; TMZ, temozolomide.

|  | **PFS** | | |  | **OS** | | |
| --- | --- | --- | --- | --- | --- | --- | --- |
| **Variable** | **HR** | **95% CI** | ***P*-value** |  | **HR** | **95% CI** | ***P*-value** |
| Age (≥ 63 years) | 0.62 | [0.40-0.99] | 0.044* |  | 1.20 | [0.76-1.89] | 0.444 |
| Sex (female) | 0.77 | [0.48-1.21] | 0.256 |  | 0.88 | [0.54-1.42] | 0.596 |
| KPS (> 80%) | 0.82 | [0.48-1.38] | 0.444 |  | 0.57 | [0.33-0.99] | 0.045* |
| TMZ consolidation (≥ 6 cycles) | 0.08 | [0.04-0.17] | < 0.001* |  | 0.31 | [0.18-0.52] | < 0.001* |
| CRTAC1 | 1.12 | [0.72-1.76] | 0.614 |  | 0.56 | [0.35-0.92] | 0.022* |
| HRG | 0.72 | [0.44-1.19] | 0.205 |  | 0.83 | [0.49-1.41] | 0.486 |
| IL1R2 | 3.06 | [1.13-8.30] | 0.028* |  | 1.73 | [0.68-4.41] | 0.252 |

**Table S7.** Relationship between serum prognostic protein levels and hematological parameters, assessed with Pearson's chi-squared test. RBC, red blood cells; WBC, white blood cells.

| **Proteins** | **RBC** | **WBC** | **Neutrophils** | **Lymphocytes** | **Monocytes** | **Platelets** |
| --- | --- | --- | --- | --- | --- | --- |
|  | **R; *Q*-value** | **R; *Q*-value** | **R; *Q*-value** | **R; *Q*-value** | **R; *Q*-value** | **R; *Q*-value** |
| **CRTAC1** | -0.19; 1.000 | -0.57; < 0.001* | -0.52; < 0.001* | -0.14; 1.000 | -0.14; 1.000 | 0.01; 1.000 |
| **HRG** | -0.20; 1.000 | -0.39; 0.002* | -0.38; 0.003* | -0.01; 1.000 | -0.11; 1.000 | 0.13; 1.000 |
| **IL1R2** | 0.05; 1.000 | 0.37; 0.005* | 0.38; 0.003* | -0.01; 1.000 | 0.06; 1.000 | -0.20; 1.000 |
